# Transcriptional insights into key genes and pathways controlling muscle lipid metabolism in broiler chickens

**DOI:** 10.1186/s12864-019-6221-0

**Published:** 2019-11-15

**Authors:** Lu Liu, Xiaojing Liu, Huanxian Cui, Ranran Liu, Guiping Zhao, Jie Wen

**Affiliations:** 1 Institute of Animal Sciences, Chinese Academy of Agricultural Sciences, Beijing, 100193 China; 2 State Key Laboratory of Animal Nutrition, Beijing, 100193 China

**Keywords:** Triglyceride metabolism, Steroid biosynthesis, Intramuscular fat, Pectoralis muscle tissue, Gene expression, Pathways, Chicken

## Abstract

**Background:**

Intramuscular fat (IMF) is one of the most important factors positively associated with meat quality. Triglycerides (TGs), as the main component of IMF, play an essential role in muscle lipid metabolism. This transcriptome analysis of pectoralis muscle tissue aimed to identify functional genes and biological pathways likely contributing to the extreme differences in the TG content of broiler chickens.

**Results:**

The study included Jingxing-Huang broilers that were significantly different in TG content (5.81 mg/g and 2.26 mg/g, *p* < 0.01) and deposition of cholesterol also showed the same trend. This RNA sequencing analysis was performed on pectoralis muscle samples from the higher TG content group (HTG) and the lower TG content group (LTG) chickens. A total of 1200 differentially expressed genes (DEGs) were identified between two groups, of which 59 DEGs were related to TG and steroid metabolism. The HTG chickens overexpressed numerous genes related to adipogenesis and lipogenesis in pectoralis muscle tissue, including the key genes *ADIPOQ*, *CD36*, *FABP4*, *FABP5, LPL*, *SCD*, *PLIN1*, *CIDEC* and *PPARG*, as well as genes related to steroid biosynthesis (*DHCR24*, *LSS*, *MSMO1*, *NSDHL* and *CH25H*). Additionally, key pathways related to lipid storage and metabolism (the steroid biosynthesis and peroxisome proliferator activated receptor (PPAR) signaling pathway) may be the key pathways regulating differential lipid deposition between HTG group and LTG group.

**Conclusions:**

This study showed that increased TG deposition accompanying an increase in steroid synthesis in pectoralis muscle tissue. Our findings of changes in gene expression of steroid biosynthesis and PPAR signaling pathway in HTG and LTG chickens provide insight into genetic mechanisms involved in different lipid deposition patterns in pectoralis muscle tissue.

## Background

With the improvement of living standards, there is a gradual increase in consumer demand for meat quality, especially in China. Meat quality is a complex concept that includes appearance, sensory, hygienic and nutritional attributes [1]. Intramuscular fat (IMF) content is commonly used in livestock and poultry industry as an indicator of meat quality influencing tenderness, color, juiciness and flavor [2–5]. Chickens with higher IMF content usually have a higher level of consumer preference.

Given the effect of lipid deposition on poultry meat, many studies have investigated the control of IMF traits in chickens. Genome-wide association analysis, polymorphism analysis and “omics” data is a common approach to identify loci and candidate genes associated with IMF [6–8]. The differential deposition mechanism of IMF in different breeds, tissues and ages has also been studied. In our previous study, the effects of breed and age on IMF deposition were explored in Beijing-you chicken and Arbor Acres, and several differentially expressed genes (DEGs) (*MYBPC1*, *CETP*, *GLTPD1* and *SNX4*) were identified for IMF developmental processes [9]. For Beijing-you and Cobb chicken, *ACADL*, *ACAD9*, *HADHA* and *HADHB* were identified as candidate biomarkers for IMF deposition [10]. Hub genes related to IMF deposition might be interfered by genetic background when the investigation involves various chicken breeds. Therefore, under the same genetic background, chickens with different IMF content are considered as a good model for studying the molecular mechanism of IMF deposition. Jingxing-Huang broiler, a high-quality chicken breed in China, has a higher capability of IMF deposition. Exploring the mechanism of IMF deposition in Jingxing-Huang chicken may contribute largely to improving meat quality and cultivating high-quality breed.

IMF is the amount of fat within muscles and consists mainly of triglycerides (TGs), but also contains phospholipids and cholesterol. As a complex trait, it is extremely difficult to accurately target key genes involved in IMF deposition. TGs, the most major component of IMF, are helpful to simplified phenotype and explore the underlying deposition mechanism of IMF. As a major factor in the regulation of energy metabolism, the synthesis and deposition of TG appear to be extremely important for energy metabolism and lipid deposition in muscle tissue [11]. Presently, although several studies have been reported on TG metabolism in chickens [12–14], little is known about the key genes and molecular mechanisms of TG metabolism in chicken pectoralis muscle tissue. In this study, 18 Jingxing-Huang female chickens with extremely different TG content were chosen for transcriptomic study aimed at identifying DEGs and investigating the underlying molecular mechanisms involved in alterations of lipid metabolism in pectoralis muscle tissue.

## Results

### Different lipid metabolism in pectoralis muscle tissue of HTG and LTG chickens

**Fig. 1:**
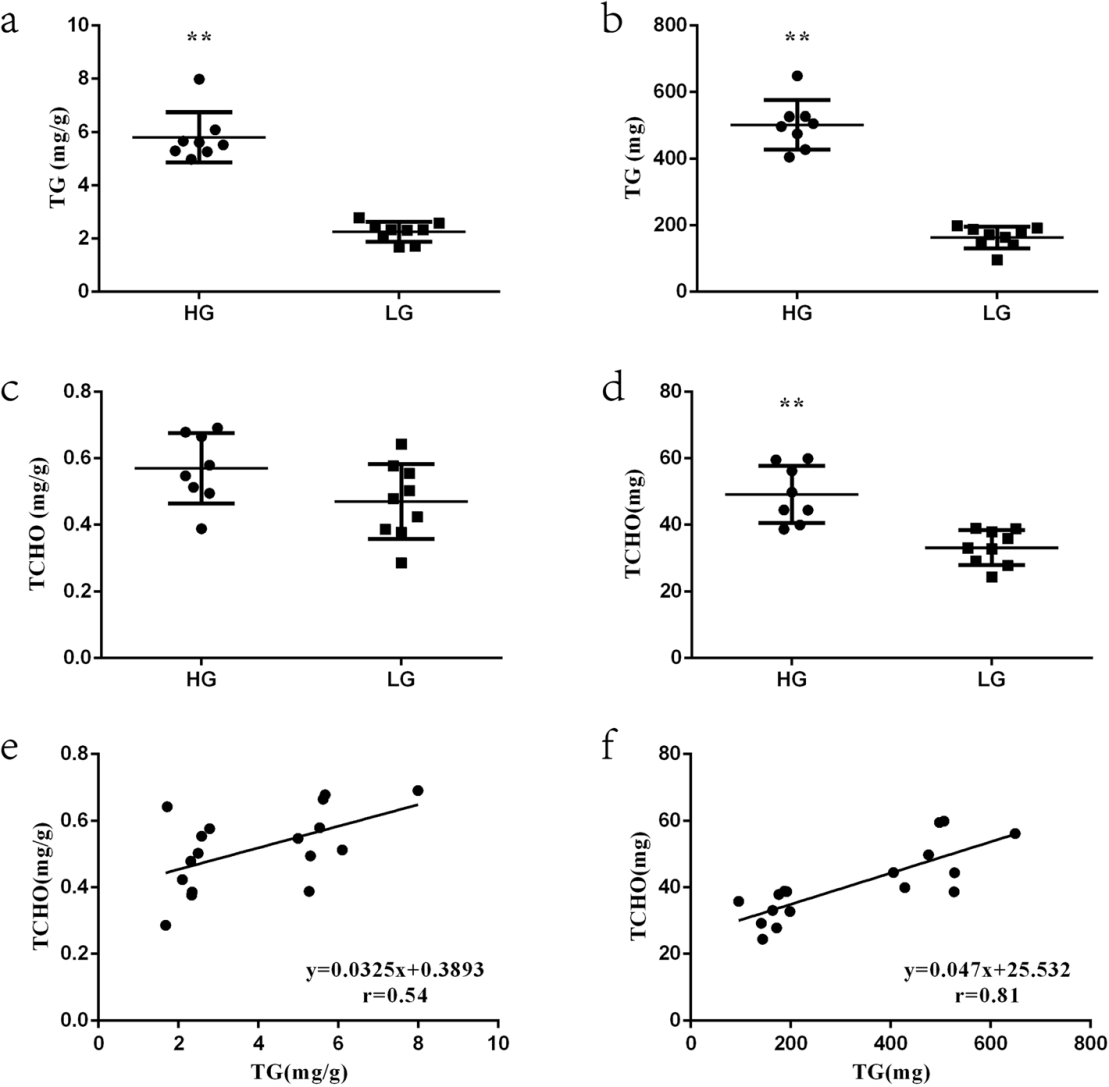
The content of triglyceride (TG) and total cholesterol (TCHO) in the higher TG content (HTG) group and lower TG content (LTG) and their correlation. **a** The relative content of TG in pectoralis muscle tissue (mg/g). **b** The absolute content of TG in pectoralis muscle tissue (mg). **c** The relative content of TCHO in pectoralis muscle tissue (mg/g). **d** The absolute content of TCHO in pectoralis muscle tissue (mg). **e** The correlation between the relative content of TG and TCHO in pectoralis muscle tissue (mg/g) was analyzed by Pearson correlation coefficient in the HTG and LTG groups (*r* = 0.54, *p* < 0.05). **f** The correlation between the absolute content of TG and TCHO in pectoralis muscle tissue (mg) was analyzed by Pearson correlation coefficient in the HTG and LTG groups (*r* = 0.81, *p* < 0.01). Data are presented as mean ± SEM (*p < 0.05 or ** *p* < 0.01)

To study lipid metabolism in pectoralis muscle tissue from HTG and LTG chickens, the relative and absolute content of TG and TCHO in pectoralis muscle tissue samples were measured. The results revealed significant differences in the TG content between chickens from the HTG and LTG groups, as shown in Fig. 1a-b. The TG content in the HTG group was extremely significantly (*p* < 0.01) higher than that in LTG group (both relative and absolute content). There was no difference (*p* > 0.05) between the two groups in the relative TCHO content, while the absolute TCHO content in the HTG group was significantly higher (*p* < 0.01) than that in the LTG group (Fig. 1c-d). The contents of TG and TCHO in pectoralis muscle tissue samples, whether the relative or absolute content, were correlated (relative content correlation, *r* = 0.54, *p* < 0.05, absolute content correlation, *r* = 0.81, *p* < 0.01), as shown in Fig. 1e-f.

### RNA sequencing data analysis

**Fig. 2:**
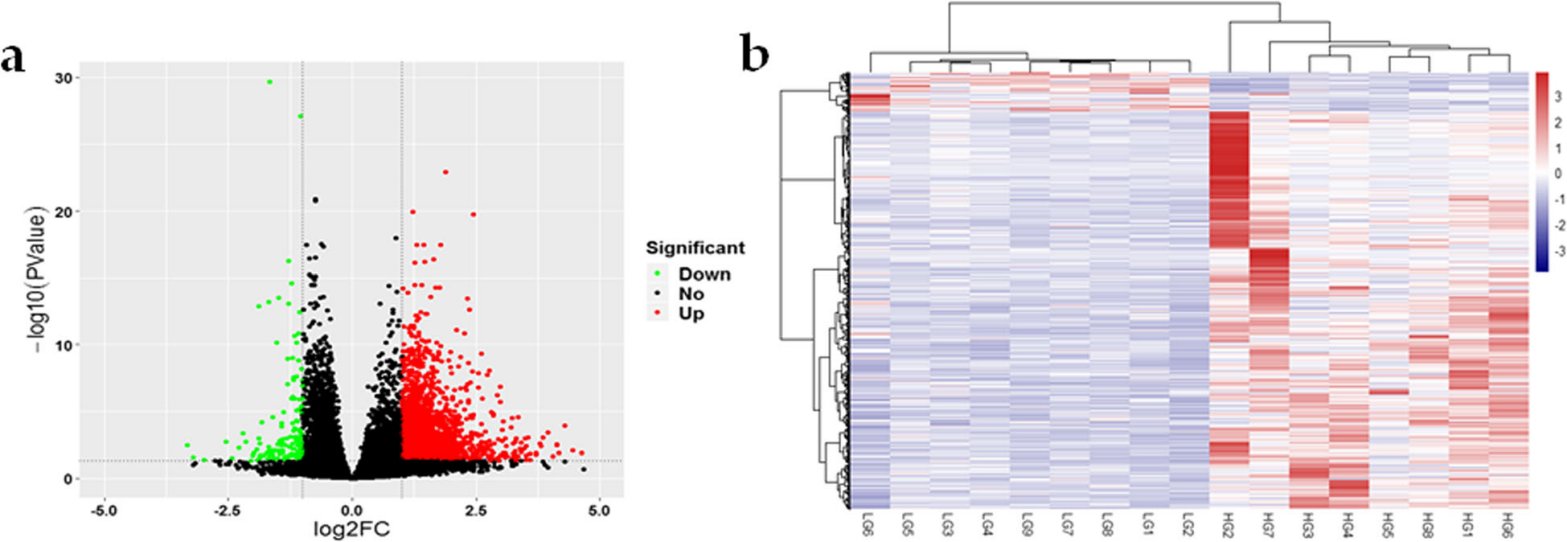
The results of the RNA sequencing analysis. **a** Volcano plot. The red dots (Up) represent significantly upregulated genes, the green dots (Down) represent significantly downregulated genes (|log2 fold change (FC)| ≥ 1 and false discovery rate (FDR) < 0.05), and the black dots (No) represent insignificantly differentially expressed genes (DEGs). **b** Hierarchical clustering analysis. Hierarchical clustering analysis was performed based on DEGs, the heat-maps of all 17 samples revealed that the gene expression profiles in same group were closely related

A total of 1200 known DEGs were identified, of which 1142 were upregulated and 58 were downregulated, in the HTG group compared with the LTG group (log2 FC ≥ 1 and FDR < 0.05), as shown in Fig. 2a and Additional file 1. One (odd-one-out) extreme individual with abnormal gene expression in the HTG group was excluded from the analysis. Hierarchical clustering analysis (based on DEGs) were performed to evaluate the consistency and variance of the samples from the HTG and LTG groups. The hierarchical clustering analysis results showed that only individuals within the same group clustered more closely (Fig. 2b).

### Identification of DEGs related to lipid metabolism of HTG and LTG group

**Fig. 3:**
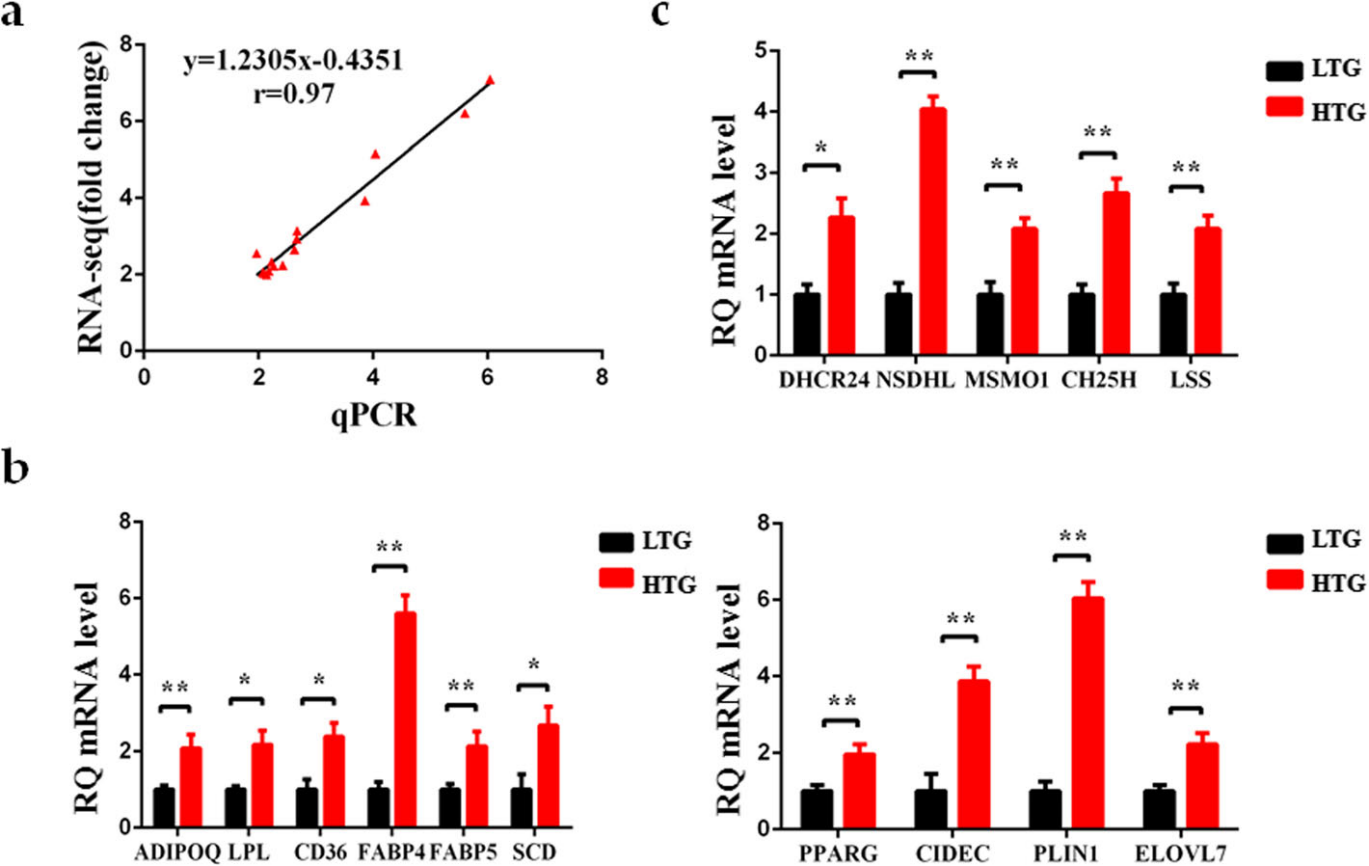
Validation of the RNA sequencing analysis data by quantitative real-time PCR (qRT-PCR) analysis. **a** Correlation analysis of the relative expression levels of 15 differentially expressed genes (DEGs) between the RNA sequencing and qRT-PCR (*r* = 0.97, *p* < 0.05). **b**-**c** Expression level of representative genes involved in TG and steroid metabolism by qRT-PCR in the HTG and LTG chickens. All genes were significantly upregulated in the HTG group compared with LTG group. Data are presented as the mean ± SEM (*p < 0.05 or ** *p* < 0.01). RQ: relative quantification

Based on 1200 known DEGs, 59 DEGs related to lipid metabolism were screened. Compared with the LTG group, 58 upregulated and 1 downregulated DEGs related to lipid metabolism were identified in the HTG group (Additional file 2), and were found to be involved in many biological processes: fatty acid binding and transport, fatty acid elongation, adipocyte differentiation, cholesterol metabolism and steroid biosynthesis. Also, almost all DEGs related to lipid deposition were significantly upregulated in the HTG group, indicating a greater capacity in lipid deposition than the LTG group. To confirm the reliability of the results, the transcript abundance of 15 key genes related to lipid metabolism were verified by qRT-PCR analysis. As shown in Fig. 3a, the fold-changes of gene expression determined by RNA-seq analysis and qRT-PCR analysis were highly correlated (*r* = 0.97, *p* < 0.05). The transcript abundance of the classical transcription factor PPARG was significantly upregulated in HTG group (*p* < 0.01). The expression of 7 genes (*ADIPOQ*, *CD36*, *FABP4*, *FABP5*, *LPL*, *SCD* and *PLIN1*) in the PPAR signaling pathway was significantly higher in the HTG group (*p* < 0.05 or *p* < 0.01). *CIDEC*, which plays an important role in controlling lipid droplet (LD) fusion and lipid storage, was significantly upregulated in the HTG group (*p* < 0.01). Additionally, *ELOVL7*, which participates in fatty acid elongation, also was significantly increased in the HTG group (*p* < 0.01) (Fig. 3b). In addition, the expression of *DHCR24*, *LSS*, *MSMO1*, *NSDHL* and *CH25H*, which are related to steroid metabolism, was significantly higher than that in the LTG group (Fig. 3c). These findings suggested that these genes are likely responsible for the higher lipid deposition in the HTG group compared with that in the LTG group.

### Functional classification and pathway enrichment of DEGs in the HTG and LTG chickens

**Fig. 4:**
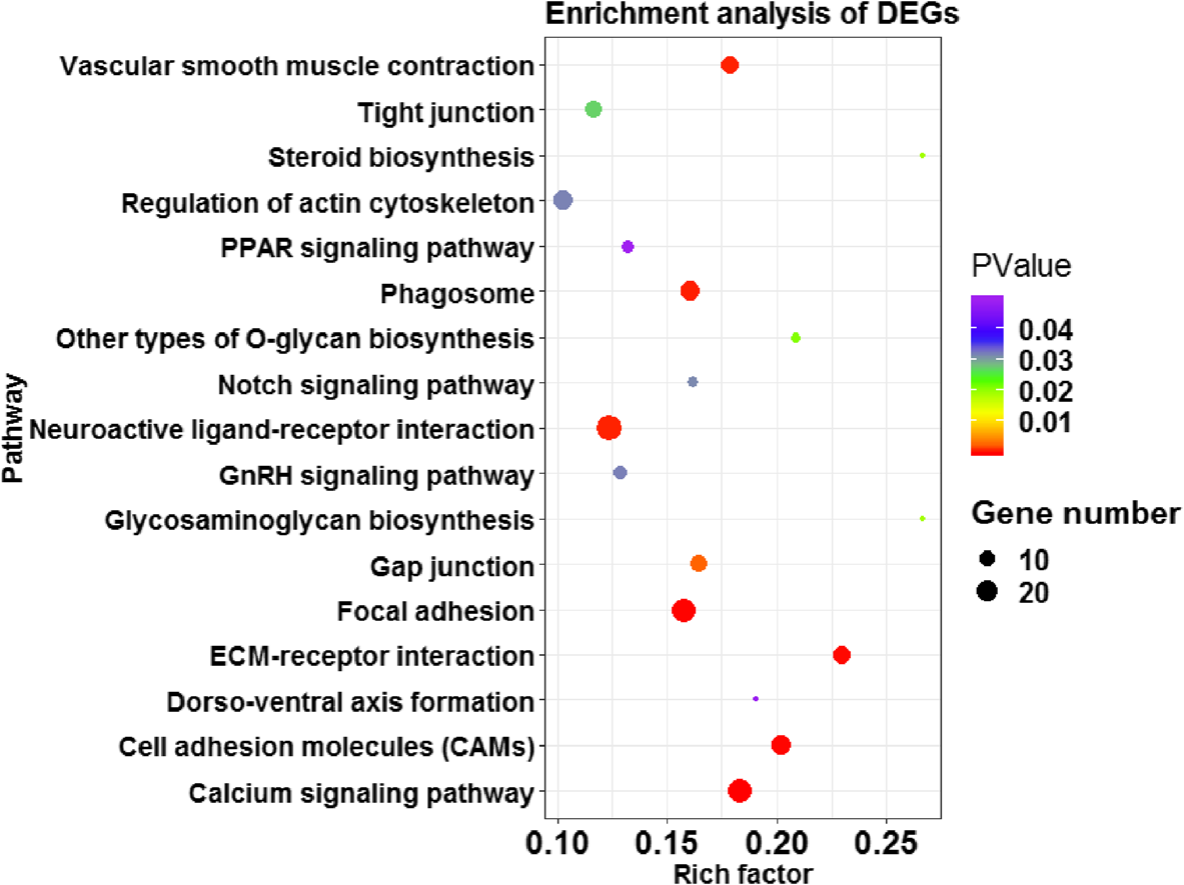
Advanced bubble chart shows significantly enriched pathways based on differentially expressed genes (DEGs) by Kyoto Encyclopedia of Genes and Genomes (KEGG) pathway analysis (*p* < 0.05). The x-axis represents rich factor (rich factor = number of DEGs enriched in the pathway/number of all genes in the background gene set). The y-axis represents the enriched pathway. Color represents enrichment significance, and the size of the bubble represents the number of DEGs enriched in the pathway

The function of the known DEGs was classified by GO enrichment analysis. A total of 55 Biological Process (BP) terms were significantly enriched (FDR < 0.05) (Additional file 3). These BP terms were mainly associated with metabolism process, muscle development, angiogenesis, signal transduction, cell activities (cell motility, migration, adhesion, communication, development and differentiation) and cytokine production. A KEGG pathway analysis was performed based on the 1200 known DEGs, and 17 pathways were significantly enriched (*p* < 0.05) (Fig. 4 and Additional file 4). Additionally, several pathways related to lipid metabolism were significantly enriched (*p* < 0.05), including steroid biosynthesis, PPAR signaling and cell junctions (focal adhesion, cell adhesion molecules, ECM-receptor interaction, gap junction, tight junction and regulation of actin cytoskeleton).

## Discussion

In recent decades, poultry meat consumption is steadily increasing worldwide [15–17]. IMF content is an important factor determining meat flavor and texture parameters for chicken meat [2–5]. An appropriate IMF content is beneficial to improve meat quality, while excessive fat content may have adverse effects, such as white striping muscle [18]. Therefore, studying the genetic mechanism of IMF deposition in muscle tissue may contribute to improving the meat quality of chicken.

Currently, it is a common way to explore candidate genes related IMF metabolism using chickens with different IMF content. Given the composition and limitation of measurement accuracy, TG content was chosen as the major phenotype instead of conventional IMF to explore the hub genes involved in lipid deposition of pectoralis muscle tissue. In this study, broiler chickens with extremely high and low TG content were used to identify the important candidate genes and pathways affecting lipid metabolism in pectoralis muscle tissue by RNA sequencing analysis.

The phenotypic analysis results indicated that the lipid metabolism processes are different between the two investigated chicken groups, including TG metabolism as well as cholesterol biosynthesis in pectoralis muscle tissue. In the present study, TG and TCHO content were correlated in both relative and absolute content. As the neutral lipid core of LD, TG and sterol esters are indispensable for LD formation [19, 20], and the amount and composition of cholesterol esters and TG can affect lipoprotein metabolism and adiposity. Therefore, it is logical to infer that increased lipid deposition in pectoralis muscle tissue is affected by TG and TCHO content. To investigate the molecular regulation of TG and steroid lipid metabolism in chicken pectoralis muscle tissue, 59 DEGs related to lipid deposition were further analyzed.

The formation of LDs is a complex process, which includes the synthesis of neutral fat as well as the formation, growth and expansion of LD. In general, there is a balance between dietary absorbed fat, de novo synthesis of fatty acids (lipogenesis) and fat catabolism involving key enzymes and transcription factors [21]. *SCD* encodes a key rate-limiting enzyme in lipogenesis, which transform palmitic acid (C16:0) and stearic acid (C18:0) to palmitoleic (C16:1) and oleic (C18:1n-9) [22]. C16:0 and C18:1, the most abundant cellular long-chain fatty acids (LCFAs), are mainly used as components of TGs [23]. In addition, very long-chain fatty acids (VLCFAs) also have unique functions in lipid metabolism. The elongation of very long-chain fatty acid (ELOVL) protein family is required for the rate-limiting step in the elongation cycle of the synthesis of LCFAs and VLCFAs [24, 25]. ELOVL7 is a newly discovered ELOVL protein family member, which triggers lipid accumulation in differentiated adipocytes [26]. Previous studies revealed that female chickens exhibited increased *SCD* expression in pectoralis muscle tissue than male chickens [27]. However, the relationship between *ELOVL7* gene and lipid deposition is still poorly understood in chicken muscle tissue. The expression of *SCD* and *ELOVL7* in the HTG group was higher than that in the LTG group, indicating that increased synthesis of fatty acids might promote the synthesis and deposition of TGs. Except for de novo synthesis and elongation of FA, the utilization of free FA is also a key step in lipid metabolism. FABP5 was found to be involved in the transport of large amounts of intracellular FAs into the nucleus to activate *PPARG* [28, 29]. Previous study indicated that upregulated *FABP5* might contribute to excessive fat deposition in domestic ducks [30]. Compared with the LTG group, the mRNA level of *FABP5* and *PPARG* was elevated in the HTG group. In addition, the upregulated expression of certain adipocyte differentiation markers, including *ADIPOQ*, *FABP4*, *LPL* and *CD36* [31–36] may be associated with increased lipid accumulation in the HTG group. CIDEC, a kind of LD-associated enzymes involved in LD fusion and growth, is mainly expressed to increase intracellular TG concentration [37]. CIDEC binds to the surface of LD and co-locates with the lipid-binding proteins, perilipins (PLINs) [38, 39]. PLIN1 was found to interact with CIDEC to promote LD formation by activating the PPARG signaling pathway [40–42]. According to published reports, higher *PLIN1* and *CIDEC* expression promoted higher fat accumulation in chickens [43]. In this study, *CIDEC* and *PLIN1* were all upregulated in the HTG group, which is consistent with their increased TG content. The upregulation of all these genes indicated a higher lipid biosynthesis in the HTG group. Previous studies have identified numerous candidate genes (*PPARs*, *FABPs*, *LPL*, *SCD*, *KLFs* and *ACSLs*) related to IMF deposition in chickens [7–10]. Most of these genes are associated with TG deposition in this study, indicating they might play an important role in IMF deposition by regulating TG metabolism.

Most of the intracellular cholesterol is positively correlated with LDs and cholesterol homeostasis may play a key role in the regulation of adipocytes size and function [44]. In this study, consistent with the phenotypic trait, the expression of key genes (*DHCR24*, *LSS*, *MSMO1*, *NSDHL* and *CH25H*), which encode proteins that are involved in steroid biosynthesis process [45–49], was upregulated in the HTG group compared with the LTG group. The quantity of fat deposition increases faster and earlier in fast-growing chickens than that in slow-growing chickens. The expression of genes involved in cholesterol biosynthesis in liver and hypothalamus tissues, such as *LSS*, *NSDHL* and *DHCR24*, was higher in the fast-growing chickens than that in the slow-growing chickens [50]. Consistent with our results, active cholesterol metabolism may be associated with increased fat deposition in chickens. Currently, reported studies mainly focused on the dietary effect on cholesterol synthesis [51] and the function of endogenous steroid metabolism in hepatic lipid deposition [52]. This study highlighted the contribution of steroid metabolism to muscle lipid metabolism and provided a new clue for exploring the mechanism of IMF deposition in chickens.

**Fig. 5:**
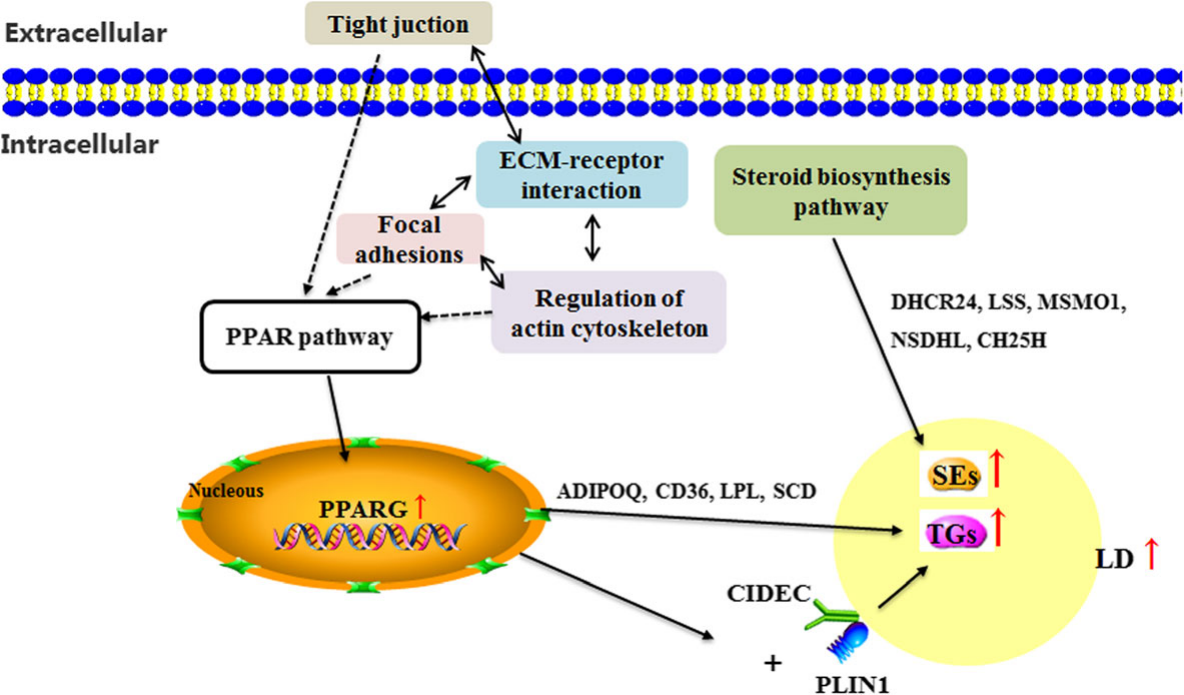
The potential regulatory network of lipid metabolism according to the differentially expressed genes (DEGs) enriched in the Kyoto Encyclopedia of Genes and Genomes (KEGG) pathways. TGs: Triglycerides; SEs: Sterol esters. Dotted arrows indicate possible regulatory relationships; solid arrows indicate reported regulatory relationships; double-ended arrows indicate bidirectional regulatory relationships

Based on the identified DEGs, KEGG pathway analysis was conducted to investigate the regulatory network underlying differential lipid deposition in chicken pectoralis muscle tissue. Among the DEGs associated with lipid metabolism, six DEGs (*ADIPOQ*, *CD36*, *LPL*, *SCD, PPARG* and *PLIN1*) were significantly enriched in the PPAR signaling pathway (*p* < 0.05). Several DEGs (*DHCR24*, *LSS*, *MSMO1*, *NSDHL* and *CH25H*) that participate in cholesterol synthesis were significantly enriched in the steroid biosynthesis pathway (*p* < 0.05). Additionally, DEGs were also significantly enriched (*p* < 0.05) in calcium signaling pathway and junction-related pathways (focal adhesions, cell adhesion, gap junction, tight junction, regulation of actin cytoskeleton and ECM-receptor interaction). Many studies have shown that the cell junction-related pathways may contribute to lipid deposition [9, 53, 54]. These results indicated that the above pathways might be the key pathways for lipid deposition in chicken pectoralis muscle tissue and a possible molecular regulatory network was constructed (Fig. 5). After activating the transcription factor PPARG in the PPAR signaling pathway, lipogenesis genes (*ADIPOQ*, *CD36*, *LPL* and *SCD*) may be upregulated to promote TG synthesis. In addition, PPARG may promote the interaction of PLIN1 with CIDEC to accelerate LD formation. At the same time, the upregulated expression of cholesterol synthesis genes (*DHCR24*, *LSS*, *MSMO1*, *NSDHL* and *CH25H*) in the steroid biosynthesis pathway may increase steroid ester synthesis. There is no doubt for the importance of PPAR signaling pathway in regulating lipid metabolism among muscle, liver and adipose tissues. However, different regulatory network centered on PPAR signaling pathway may contribute to specific lipid deposition in tissues. In this study, the active network, including PPAR signaling pathway and steroid biosynthesis pathway, might lead to an increase in lipid deposition in chicken pectoralis muscle tissue. In the future, much effort is still needed to further insight into the genetic regulation of IMF deposition in chickens.

## Conclusions

In summary, chickens from the higher TG content group (HTG) and lower triglyceride (TG) content group (HTG) were used to identify candidate genes and pathways related to differential lipid metabolism in pectoralis muscle tissue. The results showed that increased TG metabolism was accompanied by an increase of the steroid synthesis by regulating the expression of related genes (*ADIPOQ*, *CD36*, *FABP4*, *FABP5*, *LPL*, *SCD*, *PLIN1*, *PPARG*, *CIDEC*, *DHCR24*, *LSS*, *MSMO1*, *NSDHL* and *CH25H*). The results suggested that the PPAR pathway and steroid biosynthesis pathway might play a dominant role in this process. These findings provide new clues to understand revealing the molecular mechanisms underlying differential lipid deposition in chicken pectoralis muscle tissue.

## Methods

### Ethics statement

This study was conducted in accordance with the Guidelines for Experimental Animals established by the Ministry of Science and Technology (Beijing, China). All experimental protocols were approved by the Science Research Department (in charge of animal welfare issues) of the Institute of Animal Sciences, Chinese Academy of Agricultural Sciences (Beijing, China) (No. IAS2019–21).

### Animals and sampling

Jingxing-Huang female broilers were obtained from the Institute of Animal Sciences, Chinese Academy of Agricultural Sciences. All birds (*n* = 520) were raised in three-story step cages (one bird per cage) under the same recommended environmental and nutritional conditions. The basal diet was formulated based on the National Research Council (1994) requirements and the Feeding Standards of Chickens established by the Ministry of Agriculture, Beijing, China (2004).

All chickens were individually euthanized by carbon dioxide anesthesia and exsanguination by severing the carotid artery at 98 days of age after 12-h fasting (no additional feed was supplied and the feed trough was not emptied). After slaughtering, the pectoralis major muscle was dissected in the same area in all chickens. The pectoralis major muscle samples were weighed, snap-frozen in liquid nitrogen, and stored at − 80 °C for subsequent RNA isolation. The remaining pectoralis major muscle tissues were removed, weighed, and stored at − 20 °C for the measurement of TG and total cholesterol (TCHO) contents.

### Measurement of biochemical indices

The TG and TCHO contents in pectoralis muscle tissue samples were measured using TG and TCHO assay kits (Nanjing Jiancheng Bioengineering Institute, Nanjing, China). Pectoralis muscle tissue samples (about 2 g) from each chicken were homogenized with absolute ethanol at room temperature and centrifuged (1000×g, 20 min). After centrifugation, the supernatant was used for TG and TCHO measurement. A 2.5-μL aliquot of the supernatant and 250 μL reagent were co-incubated at 37 °C for 10 min. The absorbance of each sample was measured using a microplate reader at 510 nm. The assay was performed according to the manufacturer’s instructions.

### RNA extraction and sequencing

Chickens with extremely higher (HTG, *n* = 9) and lower (LTG, n = 9) TG content were used for RNA extraction and sequencing. Pectoralis muscle tissue samples from the HTG and LTG group were selected to isolate total RNA using TRIzol reagent (Invitrogen, Carlsbad, CA, USA). The detection of RNA quality was referred to in Resnyk et al. [55]. RNA purity was checked using the kaiaoK5500_®_Spectrophotometer (Kaiao, Beijing, China) and RNA integrity and concentration was assessed using the RNA Nano 6000 Assay Kit of the Bioanalyzer 2100 system (Agilent Technologies, CA, USA). After determining the concentration, purity and integrity, the RNA samples with an A260/A280 ratio between 1.8 and 2.0 and an RNA integrity number > 7.5 were used for RNA sequencing and quantitative real-time PCR (qRT-PCR) analysis.

We used the methodology of cDNA library construction previously described by Chen et al. [56]. The mRNA was enriched by binding of the mRNA poly-A tail to magnetic beads with Oligo (dT) and fragmented into small pieces. Single strand cDNA and double strand cDNA were synthesized using mRNA as a template. The double-stranded cDNA was purified using the QIAQuick PCR purification kit (QIAGEN, Valencia, CA, USA). After purification, end repair, and ligation to sequencing adapters, agarose gel electrophoresis was used for fragment size selection. Finally, PCR enrichment was performed to obtain the final cDNA library. RNA-sequencing was performed on an Illumina NovaSeq 6000 S2 (Illumina, San Diego, CA, USA) by Annoroad Genomics (Beijing, China) and 150-bp paired-end reads were generated (Additional file 5).

### Data analysis of RNA sequencing

Sequence adapters and low-quality reads (read quality < 30) were removed by Trimmomatic (v0.32), and quality control checks on raw sequence data were performed with FastQC. Sequencing reads were mapped to the chicken reference genome [Ensembl GRCg6a (GCA_000002315.5)] using the HISAT2 program [57]. To quantify the expression of each transcript, alignment results were analyzed by the Cufflinks (v2.0.2) software [58]. Analysis of differential expression of transcripts was performed with DESeq2 package (v 1.24.0). Genes with false discovery rate (FDR) value < 0.05 and |log2 fold change (FC)| ≥ 1 were considered to be DEGs.

Hierarchical clustering analysis was performed to determine the variability and repeatability of the samples and a volcano plot was used to visualize the overall distribution of DEGs. Gene ontology (GO) enrichment analysis was performed to identify the gene function classes and categories of DEGs using the DAVID functional annotation clustering. Kyoto Encyclopedia of Genes and Genomes (KEGG) pathway enrichment analysis was performed by KOBAS 3.0 [59] (http://kobas.cbi.pku.edu.cn). The significance level for GO terms and the KEGG pathway was set with FDR < 0.05 and *p* < 0.05, respectively.

### Quantitative real-time PCR (qRT-PCR) analysis

All PCR primers were designed at or just outside exon/exon junctions to avoid the amplification of residual genomic DNA using the Primer-BLAST on the NCBI website, and specificity was determined using BLASTN (Additional file 6).

qRT-PCR analysis was performed after a reverse transcription reaction. cDNA was prepared with 2.0 μg of total RNA of each sample, by reverse transcription using the FastQuant RT Kit (Tiangen, Beijing, China) in accordance with the kit manufacturer’s instructions. Each PCR mixture was prepared in a final volume of 20 μL consisting of 10 μL of 2 × iQTM SYBR Green Supermix, 0.5 μL (10 mmol/L) of each primer, and 1 μL of cDNA. Samples were amplified on the QuantStudio 7 Flex system (Applied Biosystems, Shanghai, China) using 40 cycles (95 °C for 3 min, 95 °C for 3 s and 60 °C for 34 s). The amplification procedure was performed with 3 replicates for each sample. The collected data were analyzed using the 2^−ΔΔCT^ method [60], and all the results were normalized to the *18S* rRNA gene.

### Statistical analyses

Significance of differences between groups was tested by the Student *t*-test using the SPSS Version 22.0 (IBM Corp, Armonk, NY, USA). Confidence limits were set at 95% and *p* < 0.05 (*) or *p* < 0.01 (**) was considered significant. Data are presented as the mean ± standard error (SEM).

## Supplementary information

**Additional file 1.** The screened DEGs between HTG and LTG group.

**Additional file 2.** The 59 DEGs related to lipid metabolism.

**Additional file 3.** The enriched GO-terms based on 1200 DEGs.

**Additional file 4.** The enriched pathways based on 1200 DEGs.

**Additional file 5.** The details about RNA-seq data statistics in this study.

**Additional file 6.** The specific primers for qPCR in this study.

## Data Availability

The RNA sequencing clean data reported in this paper have been deposited in the Genome Sequence Archive [61] in BIG Data Center [62] under accession number CRA001908 which can be publicly accessed at http://bigd.big.ac.cn/gsa.

## Abbreviations

*ADIPOQ*: Adiponectin
BP: Biological Process
*CH25H*: Cholesterol 25-hydroxylase
*CIDEC*: Cell death-inducing DNA fragmentation factor subunit alpha (DFFA)-like effector c
DEGs: Differentially expressed genes
*DHCR24*: 24-Dehydrocholesterol reductase
*ELOVL7*: Fatty acid elongase 7
ELOVLs: Fatty acid elongases
*FABP4*: Fatty acid binding protein 4
*FABP5*: Fatty acid binding protein 5
FABPs: Fatty acid binding proteins
FC: Fold change
FDR: False discovery rate
FFA: Free fatty acid
GO: Gene Ontology
HTG: Higher TG content
IMF: Intramuscular fat
KEGG: Kyoto Encyclopedia of Genes and Genomes
LCFAs: Long-chain fatty acids
LD: Lipid droplet
*LPL*: Lipoprotein lipase
*LSS*: Lanosterol synthase
LTG: Lower TG content
*MSMO1*: Methylsterol monooxygenase 1
*NSDHL*: NAD(P) dependent steroid dehydrogenase-like
PLINs: Perilipins
PPAR: Peroxisome proliferator-activated receptor
qRT-PCR: quantitative real-time PCR
*SCD*: Stearoyl-CoA desaturase
TCHO: Total cholesterol
TG: Triglyceride
VLCFAs: Very long-chain fatty acids
WAT: White adipose tissue
